# Synthesis of single-crystal low-loss LiB_3_O_5_ nanowire and its optical properties

**DOI:** 10.1038/srep39389

**Published:** 2016-12-19

**Authors:** Guang-Yuan Qu, Cheng-Wei Yang, Guo-xin Cui, Xue-Jin Zhang, Yan-Qing Lu

**Affiliations:** 1 National Laboratory of Solid State Microstructures, Collaborative Innovation Center of Advanced Microstructures and College of Engineering and Applied Sciences, Nanjing University, Nanjing 210093, China

## Abstract

Optical-quality single-crystal LiB_3_O_5_ (LBO) nanowires are synthesized for the first time using a sol–gel method. The LBO nanowires possess diameters ranging from 200 to 800 nm and lengths of up to 200 μm, and exhibit excellent uniformity, smooth surfaces, and good mechanical properties. A typical propagating loss of 0.038 dB/μm at 532 nm is obtained for a 620 nm-diameter nanowire. This is a decrease of one order of magnitude compared with that of a β-BaB_2_O_4_ (BBO) nanowire with similar diameter, which makes the LBO nanowire a promising candidate to construct miniaturized nonlinear photonic devices.

During the last several decades, many borate-based nonlinear optical (NLO) crystals have been discovered, which have greatly expanded the range of laser wavelengths from the near infrared (NIR) through the visible to the ultraviolet (UV), and deep-UV spectral regions^1^,^2^,^3^,^4^. Among current NLO crystals, lithium triborate (LiB_3_O_5_, LBO) developed in 1989 by Chen and co-workers^5^ is one of the most important. LBO with orthorhombic symmetry crystallizes in space group *P*_na21_, and has lattice parameters *a* = 8.4473 Å, *b* = 7.3788 Å, and *c* = 5.1395 Å. It has favorable NLO properties such as high optical quality, broad transparency range (160 nm–2.6 μm), moderate NLO coefficient, wide acceptance angle, small walk-off angle, good mechanical properties and high chemical stability^5^. As a consequence, LBO crystals have been widely used in harmonic generations of high-power pulsed lasers in the NIR, visible and UV regions, as well as optical parametric oscillators and amplifiers, and dominate the market of NLO borate crystals^6^,^7^,^8^.

On the other hand, one-dimensional nanowires also have attracted growing interest in recent years. Their unique properties and applications make them superior to their bulk counterparts^9^,^10^,^11^. Among them, NLO crystal nanowires have received considerable attention due to the practical use of its nonlinear properties in frequency converter, subwavelength microscopy and other micro-/nanophotonic devices^12^,^13^,^14^. Compared with bulk crystals, NLO nanowires can offer tight optical confinement with dense field intensity. The excellent waveguiding capability further enhances the light–matter interactions, which may improve its nonlinear characteristics greatly. Recently, much progress has been made in the synthesis and applications of NLO crystal nanowires such as single-crystalline β-BaB_2_O_4_ (BBO), LiNbO_3_, KNbO_3_ and NaNbO_3_ nanowires and nanorods^12^,^13^,^14^,^15^,^16^. Broadband waveguiding capability and efficient second harmonic generation have been demonstrated. These advances reveal the potential of NLO crystal nanowaveguides for efficient frequency conversion. Despite these exciting developments, LBO nanomaterials have hardly been explored. The conversion efficiency of the second harmonic generation of LBO is 3 times higher than that of KH_2_PO_4_ (KDP). The surface damage threshold of LBO is 3.5 times higher than that of KDP and 1.6 times higher than that of BBO, which is the highest one among current NLO crystals measured so far^5^. This remarkable feature of LBO over its peers warrants broad prospects for its applications in nanoscale nonlinear photonics. Therefore, growing LBO nanowires, investigating their optical properties and application should be of great interests and benefit, and even may lead to the development of other new NLO crystal nanowires.

## Results

### Synthesis of single-crystal LBO

**Figure 1:**
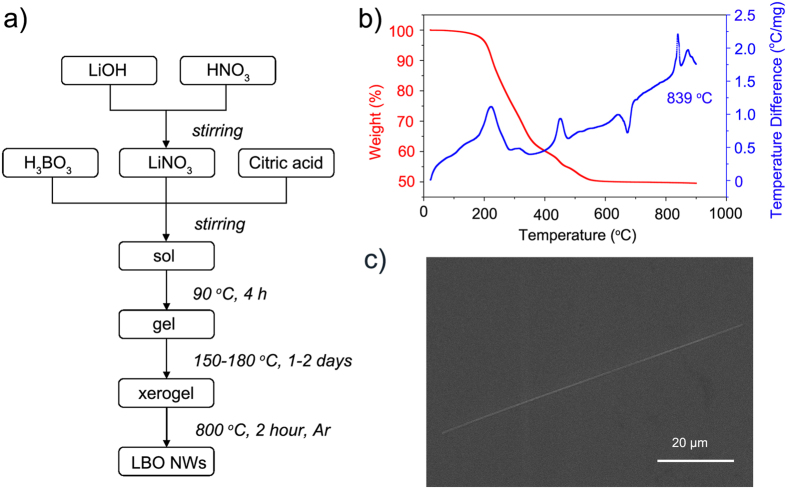
(**a**) Synthesis of LBO nanowires. (**b**) DSC-TGA curve of the xerogel powder. (**c**) FE-SEM image of a typical LBO nanowire with a diameter of 400 nm and length of 85 μm.

A sol–gel method followed by a post annealing procedure was used to synthesize single-crystal LBO nanowires. The process of sol-gel is illustrated in Fig. 1a, and detailed description please refer to the **Methods** part of this paper.

To determine the annealing temperature of the brown xerogel, the powder was characterized by a SETARAM Labsys16 differential scanning calorimetry–thermogravimetric analysis (DSC-TGA). As illustrated in Fig. 1b, the DSC-TGA curve contains a peak around 839 °C, which is consistent with the melting point of LiB_3_O_5_. Therefore, 800 °C should be a suitable temperature to anneal the xerogel powder. So the xerogel was placed in a crucible and heated in a muffle furnace at 800 °C for 2 h. An argon flow of 100 sccm was passed through the quartz tubes during annealing. After annealing, loose powder with a decreased volume and weight compared with those of the xerogel was obtained. The solid powder was washed with deionized water and alcohol several times to remove non-oxidized carbon residues, and finally dried at 60 °C in air.

A Hitachi S-3400N II field-emission scanning electron microscopy (FE-SEM) was used to examine the as-synthesized products. The obtained nanowires have a diameter of 200 to 800 nm and length of 40 to 200 μm. An FE-SEM image of a typical nanowire with a diameter of 400 nm and length of 85 μm is shown in Fig. 1c.

### Characterization of LBO with X-ray powder diffraction (XRD)

**Figure 2:**
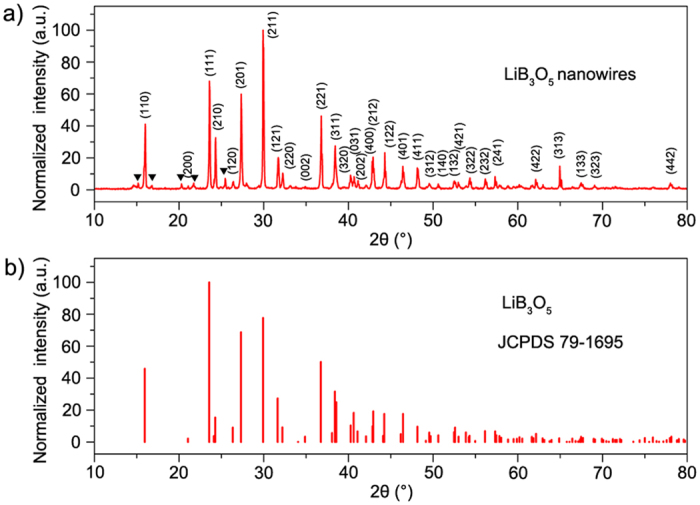
XRD pattern of (**a**) as-synthesized LBO nanowires and (**b**) LiB_3_O_5_ (JCPDS file no. 79-1695).

XRD on a Rigaku Ultima III system with high-intensity Cu K_*α*_ radiation was used to examine the crystal structure and phase composition of the nanowires; the results are presented in Fig. 2a. The XRD pattern coincides well with the Joint Committee on Powder Diffraction Standards (JCPDS) file No. 79-1695 (Fig. 2b), from which the nanowires can be indexed appropriately as orthorhombic LiB_3_O_5_. The fitting crystalline parameters are *a* = 8.4473 Å, *b* = 7.3788 Å, and *c* = 5.1395 Å. The peaks marked by triangle symbols indicate the existence of the Li_3_(B_7_O_12_) (JCPDS file No. 80-0672) as an impurity. The refinement of as-synthesized products ensures the relatively pure nanowires for subsequent structural and optical characterization.

### Characterization of LBO with FE-SEM and TEM

**Figure 3:**
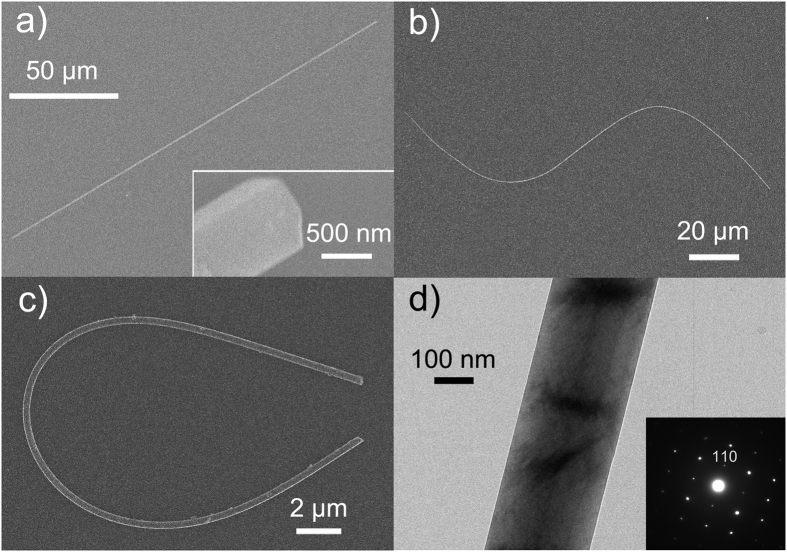
FE-SEM images of typical LBO nanowires with (**a**) a diameter of 710 nm and length of 194 μm, (**b**) a diameter of 300 nm and length of 178 μm and an “S” shape, and (**c**) a diameter of 350 nm and length of 37 μm and a “U” shape. The inset of (**a**) shows a close-up image of the end of the nanowire with a perfect ridge end face. (**d**) Low-magnification TEM image of a single LBO nanowire with a diameter of 265 nm. The inset shows its corresponding SAED pattern.

The morphology and microstructure of the as-synthesized LBO nanowires were further characterized by means of FE-SEM and transmission electron microscopy (TEM) on a JEOL JEM-2100 system, as illustrated in Fig. 3. An FE-SEM image of a typical LBO nanowire with a diameter of 710 nm and length of 194 μm, corresponding to a length-to-width aspect ratio of about 270, is provided in Fig. 3a. The close-up FE-SEM image shown in the inset of this figure reveals the rectangular cross-section and perfect ridge end face of the nanowire. An “S”-shaped LBO nanowire (diameter = 300 nm) and a “U”-shaped LBO nanowire (diameter = 350 nm) modeled by a nanofiber taper are presented in Fig. 3b and c, respectively. Compared with the “S”-shaped LBO nanowire, the “U”-shaped LBO nanowire is smooth and flexible even though it is only 37 μm long. The easy shaping and good mechanical properties of LBO nanowires make them promising as 1D building blocks for various complicated nanoscale nonlinear photonic devices.

A low-magnification TEM image of a single LBO nanowire with a diameter of 265 nm is shown in Fig. 3d. The LBO nanowire exhibits excellent diameter uniformity and high surface smoothness. The inset shows the corresponding selected-area electron diffraction (SAED) pattern, which confirms that the nanowire is single crystalline. The lattice spacing along the axial direction determined from the SAED pattern is 0.544 nm, which can be indexed as the [110] direction according to the d value obtained from the XRD results in Fig. 2. It is well known that many crystalline materials usually exhibit an anisotropic preferential growth direction. In the case of LBO, the largest spacing between crystal planes is 0.556 nm and occurs in the [110] direction^17^, which is consistent with the lattice spacing of 0.544 nm determined from the SAED pattern. The exposed [110] crystal plane has the lowest surface energy of the planes of LBO, so the nanowires intent to grow along the [110] direction.

### The measurement on the optical propagation loss of LBO nanowire

The NLO crystal nanowires are good candidates as building blocks in micro-/nanophotonic integrated circuits. Although our LBO nanowire shows favorable microscopic profiles, the optical proprieties should be characterized to ensure their functionality. For this purpose, high-quality optical waveguides with low propagation loss are prerequisite for corresponding devices. Since bulk LBO crystals have been widely used in second harmonic generation of 1064 nm Nd:YAG lasers, here we chose 532 nm to measure the loss in a single LBO nanowire waveguide under a microscope.

**Figure 4:**
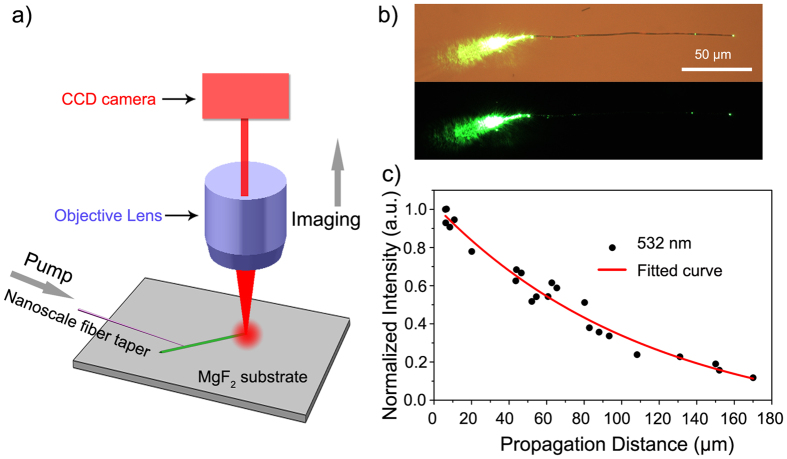
(**a**) Schematic illustration of the experimental setup for optical coupling and loss measurement of a single LBO nanowire. (**b**) Bright-field and dark-field optical microscopy images of 532-nm light transmitting through an LBO nanowire with a diameter of 620 nm and length of 172 μm. (**c**) The propagation-distance-dependent normalized output intensities of the LBO nanowire used in (b) at 532 nm. The solid curve shows the fitting of the experimental results.

The experimental setup for the optical coupling and loss measurement in a single LBO nanowire is schematically illustrated in Fig. 4a. LBO nanowires were transferred to the polished surface of the MgF_2_ substrate using a nanoscale fiber taper. The fiber taper was drawn from a standard optical fiber (Corning SMF-28) using a flame-heated drawing technique^18^. To efficiently launch light into a single LBO nanowire, we used the evanescent coupling technique through the nanoscale fiber taper mounted on a 3D moving stage. The output signal from the LBO nanowire was collected through an objective lens and then imaged by a calibrated charge-coupled-device (CCD) camera. All the measurements were performed at room temperature.

Figure 4b presents bright- and dark-field optical microscopy images of 532-nm light coupling from a fiber taper to a LBO nanowire with a diameter of 620 nm and length of 172 μm. Light was coupled into and guided along the axial direction of the LBO nanowire. Evident output was scattered from the end, and almost no scattering was observed along the entire length of the nanowire, demonstrating the excellent optical waveguiding capability of the LBO nanowire.

A propagation-distance-dependent output measurement was used to evaluate the propagation loss of the LBO nanowire^14^,^19^,^20^,^21^. The angle and position of the fiber taper were carefully adjusted so that the coupling was optimized to give maximum output from the distal end of the LBO nanowire. To keep the coupling efficiency constant, the fiber taper was horizontally moved along the axial direction of the LBO nanowire without changing the coupling angle between the fiber taper and nanowire. The output images of the end of the LBO nanowire at different propagation distances were recorded using the CCD camera without saturation. Then, we selected a 50 × 50 pixel area of the captured image with the output spot in the center and transformed the output image from a RGB image to gray level information using Adobe Photoshop. The normalized output intensity was finally obtained by summing up the gray values.

Light- and dark-field images were obtained with different launching positions of the fiber taper (Fig. 4b). The propagation distance of the LBO nanowire was measured from the light-field image while the output intensity was obtained using the dark-field image. Generally, the output intensity *I*(*x*) of a nanowire exponentially decreases along the axial length as


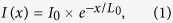


where *I*_0_ is the initial intensity, *x* is the local position along the length, and *L*_0_ is the propagation length. The propagation loss *α* is inversely proportional to *L*_0_,


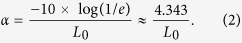


Both *L*_0_ and *α* can be obtained by measuring the output intensity of the LBO nanowire at different *x*. Figure 4c displays the propagation-distance-dependent normalized output intensities of the same LBO nanowire as in Fig. 4b at 532 nm along with the solid fitting result. According to the obtained *L*_0_ of 114.5 μm at 532 nm, the calculated *α* was 0.038 dB/μm.

## Discussion

Compared with our previous work on NLO crystal nanowires (*α* = 0.30 dB/μm at 532 nm for a BBO nanowire with a diameter of 580 nm)^14^, the propagation losses of the LBO nanowire obtained here are decreased by one order of magnitude. This indicates the LBO nanowire waveguide has better propagating properties than the BBO nanowire waveguide. The low loss of the LBO nanowire investigated here originates from its smooth surface. Our results indicate that LBO nanowire waveguides synthesized using the sol–gel method exhibit excellent propagating qualities. Using LBO nanowires as optical waveguides will enable us to obtain high light intensities with a moderate power and make it possible to extend the practical use of its nonlinear properties in micro-/nanophotonic integrated devices.

The propagation loss of the LBO nanowires in this work is still relatively high in comparison with the absorption loss in LBO bulk crystals, which is 5 × 10^−3 ^cm^−1^ (0.25 μm< *λ* *<*2.5 μm)^22^. This should be attributed to the nanoscale imperfect surface uniformity of the wires compared with that of well-polished bulk crystals. The propagating light leaking into the substrate can also cause some extra loss. To suppress the light propagation attenuation, the fabricating and measuring techniques could be improved. A larger nanowire diameter is desired as the loss decreases with increasing diameter^21^. In addition, we may select longer operation wavelength according to previous work, where lower loss were obtained in the red and infrared bands^14^,^20^,^21^. Since at least two wavelengths should be involved for nonlinear processes, the parametric down-conversion applications may even have lower general losses than that of frequency doubling. As frequency down-conversion is a fundamental solution for many classical and quantum light sources, we believe our LBO nanowire would find more exciting applications in future miniaturized nanophotonic devices.

In summary, we fabricated optical-quality single-crystal LBO nanowires for the first time using a sol–gel method assisted by post annealing. The resulting LBO nanowires possessed diameters ranging from 200 to 800 nm and lengths of up to 200 μm. They displays excellent uniformity, smooth surfaces, good shaping and easy handling properties. Based on a propagation-distance-dependent output measurement, a typical propagation loss of 0.038 dB/μm at 532 nm was obtained in a LBO nanowire with a diameter of 620 nm. It is one order of magnitude lower than that of BBO nanowires with a diameter of 580 nm (0.30 dB/μm at 532 nm). Such optical propagation characteristics of LBO nanowires make them promising elements to build miniaturized photonic devices such as nanoscale frequency-converters and nonlinear optical-modulators.

## Methods

### Recipe of sol-gel process to synthesis LBO nanowire

Commercial high-purity LiOH·H_2_O (0.05 mol), H_3_BO_3_ and HNO_3_ (65%) with a molar ratio of 1:3:1 were separately dissolved in deionized water (15 ml) to form clear solutions. As illustrated in Fig. 1a, LiOH and HNO_3_ solutions were mixed and stirred thoroughly to form a LiNO_3_ solution as shown in Eq. (3). H_3_BO_3_ solution was then added into the generated LiNO_3_ solution, as shown in Eq. (4). An appropriate amount of citric acid was added to improve the conformation of the sol. Citric acid was added also to promote the formation of LBO nanowires. After continuous stirring for about 4 h at 90 °C, a homogeneous white gel was obtained. The white gel was dried at 150–180 °C in an oven for 1–2 days, resulting in a brown xerogel.


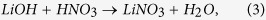



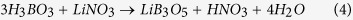


## Additional Information

**How to cite this article**: Qu, G.-Y. *et al*. Synthesis of single-crystal low-loss LiB_3_O_5_ nanowire and its optical properties. *Sci. Rep.* **6**, 39389; doi: 10.1038/srep39389 (2016).

**Publisher's note:** Springer Nature remains neutral with regard to jurisdictional claims in published maps and institutional affiliations.
